# The Impact of Gratitude on Connection With Nature: The Mediating Role of Positive Emotions of Self-Transcendence

**DOI:** 10.3389/fpsyg.2022.908138

**Published:** 2022-06-16

**Authors:** Li Chen, Jiangxin Liu, Lezhen Fu, Chang Guo, Yuhuan Chen

**Affiliations:** School of Psychology, Northwest Normal University, Lanzhou, China

**Keywords:** gratitude, connection to nature, self-transcending positive emotions, pro-social, environment

## Abstract

Gratitude, as one of the positive emotions associated with self-transcendence, is also a moral and pro-social emotion with a pro-social nature. Therefore, in order to verify whether gratitude has the same effect as pro-social in promoting connection with nature, this study (*N* = 890) divided subjects into three groups (gratitude, recreation, and control) and used a questionnaire to explore the effects of gratitude on positive emotions of self-transcendence, connection with nature, and pro-environmental tendencies (willingness to participate in environmental protection, willingness to sacrifice for the environment). The results found that (1) positive emotions of self-transcendence partially mediated the effect of the gratitude condition on connection to nature, and (2) positive emotions of self-transcendence and connection to nature were fully and continuously mediated, suggesting that the gratitude condition had an indirect effect on both (a) willingness to participate in environmental protection and (b) willingness to sacrifice for the environment. These findings imply that we may need to pay more attention to the connection between gratitude and nature to promote a harmonious relationship between humans and nature.

## Introduction

With rapid economic development, people’s living standards have improved considerably, but the pursuit of a quality of life has been accompanied by far greater pressure on the environment than before, making environmental protection an urgent issue in current social development. Over the past two decades, there has been a commitment to environmental awareness and attitudes, and engagement with the environment is predicated on the need for people to feel part of nature (Leopold, 1949). It has been noted that connection to nature is considered a key determinant of environmental attitudes (Mackay and Schmitt, 2019). In other words, people who are more connected to nature also have more environmental attitudes and behaviors (Kals et al., 1999; Mayer and Frantz, 2004; Hinds and Sparks, 2008; Davis et al., 2009). It has been suggested that the degree of connectedness to nature greatly reflects the degree of connectedness to others (Davis et al., 2009; Mayer et al., 2009), i.e., social connectedness can facilitate connectedness to nature. Whereas self-transcendent emotions as determinants of interpersonal interactions are highly influential (Goetz et al., 2010; Piff et al., 2015), individuals in a state of gratitude are even more capable of individuals in a state of gratitude are more likely to be motivated by strong social and environmental ties (Naito et al., 2010). Thus people have started to focus on the connection with nature and its role in promoting environmental behavior, starting with contact with the natural environment. But there are few experimental studies that specifically address the causal impact of the connection to nature beyond contact with nature itself (Moreton et al., 2019). This study will explore whether gratitude, one of the positive emotions of self-transcendence, can also draw people closer to their connection with nature and thus enhance environmental intentions.

## Research Background

### Connectedness to Nature and Connect With Others

Humans are not an entity that exists independently of anything else; the innate need to belong makes them exist in relationships (Adler, 1927; Freud, 1989), and the maintenance of relationships is one of the powerful motivations for achieving the need to belong (Maslow, 1962; Buss, 1990; Baumeister and Leary, 1995). In social psychology, people are concerned with social connections between people; in ecological psychology, people are concerned with connections between people and nature. The biophilia hypothesis assumes that people have a biologically based need to connect and feel connected to the wider natural world (Wilson, 1984; Kellert and Wilson, 1993; Kellert, 1997). Roszak (1995) argues that this sense of belonging extends beyond our urban sphere to include a sense of belonging to the natural world. As early as 1984, Wilson noted that disconnection from nature would be highly detrimental to people’s psychological functioning (Louv, 2005; Baxter and Pelletier, 2019), and that a strong connection to nature would not only enhance individual well-being, but also life satisfaction (Capaldi et al., 2014), vitality, and many positive effects (Capaldi et al., 2014).

The human-nature relationship has been defined in many past studies in different ways, such as connection to nature (Mayer and Frantz, 2004), relationship with nature (Nisbet et al., 2009), love and care for nature (Perkins, 2010), integration into the natural self (Schultz, 2002) and commitment to nature (Davis et al., 2009), among others. In this study, they are collectively referred to as connection to nature – the degree to which a person feels connected to nature (Schultz, 2001; Mayer and Frantz, 2004). Connection to nature is an important determinant of environmental behavior (Cialdini et al., 1997), and people who are more connected to nature have more environmental attitudes and behaviors (Kals et al., 1999; Mayer and Frantz, 2004; Hinds and Sparks, 2008; Davis et al., 2009). In turn, empirical research supports the idea that a connection to nature is a strong predictor of supporting environmental attitudes and behaviors (Mackay and Schmitt, 2019). Schultz (2001) found that subjects who incorporated nature into their self-concept held more environmentally friendly beliefs and engaged in more pro-environmental behaviors (Tam et al., 2013). This is because when one feels a part of nature, the urge to protect it is as strong as protecting oneself (Leopold, 1949).

Connecting with others can foster a connection with nature. As Mohandas Gandhi once said: “What we do to nature is actually a mirror of what we do to ourselves and to each other,” meaning that people can see how they will treat the natural world by how they treat the people around them are interconnected. It has been shown that the degree of connection to nature greatly reflects the degree of connection to others (Davis et al., 2009; Mayer et al., 2009) and that there is a moderate correlation between them (*r* = 0.44; Lee et al., 2015). Second, there is ample research that provides a basis for positive emotions to promote social connectedness. Cohn (2016) noted that people can translate their positive emotions into the ability to make positive social connections with others, with findings indicating that subjects randomly assigned to experience positive emotions exhibited greater social engagement (Isen, 1970), social inclusion (Dovidio et al., 1995), individualized other concerns (Johnson and Fredrickson, 2005), self-expression (Cunningham, 1988), interpersonal trust (Dunn and Schweitzer, 2005), perspective taking, and empathy (Nelson, 2009). Social connection is also an important determinant of positive emotions (Baumeister and Leary, 1995). Thus, the two-way hypothesis between connection to nature and positive emotions is proposed and tested through social connection (Moreton et al., 2019). Connection to nature can lead to positive emotions (Capaldi et al., 2014) and positive emotions can also enhance connection to nature (Moreton et al., 2019), with self-transcending positive emotions as a specific category of positive emotions being highly effective in inducing connection to nature (Stellar et al., 2017; Moreton et al., 2019).

### Positive Emotions of Self-Transcendence Promote Connection With Others

Positive emotions encompass all positive emotional experiences and can be divided into general positive emotions and self-transcendent positive emotions in terms of the object of the emotional experience. In this study, positive emotions in general are defined as emotional experiences and evaluations of the self, which simply evoke intrinsic emotions of the individual; self-transcending positive emotions are defined as emotional experiences and evaluations of individuals outside the self, also known as emotions that praise others. This includes inspiration, awe, gratitude, moving, respect, admiration, humility, and love (Moreton et al., 2019), although this classification is not fully identified (Keltner and Haidt, 2003; Stellar et al., 2017). Among them, self-transcendence represents the beauty and nobility of humanity. It can inspire countless others to rise above their petty, selfish concerns in the service of something greater than themselves. However, this great transformation requires more than individual effort. It also requires a collective effort to work together to create a more just and compassionate society (Wong et al., 2021). Self-transcendence is often associated with religious experiences. For example, James (1902/2004) wrote: “The only thing that religious experience, as we have studied it, unequivocally testifies to is that we can experience union with something larger than ourselves and in that union find our greatest peace.”(Wong et al., 2021). Self-transcendent emotions refer to a category of emotions, such as awe, love, promotion and appreciation that connect people in social relationships (Stellar et al., 2017). And this emotion broadens and builds one’s mindset and social resources (Fredrickson, 2001) and has the ability to bond individuals together in a way that is enjoyable for both parties (Smith and Ellsworth, 1985; Kringelbach and Berridge, 2017).

This type of emotion binds individuals in social relationships together to form groups by facilitating cooperation (Haidt, 2003) and has the ability to encourage individuals to look beyond their own momentary needs and desires and focus on the needs and desires of others (Stellar et al., 2017). In other words, the emotion of self-transcendence usually stems from focusing on the assessment of others, shifting attention to the needs and concerns of others rather than oneself. For example, empathy is an evaluation of another’s feelings of pain, gratitude is due to the generosity of others, etc. (Stellar et al., 2017). In contrast, positive emotions in general are evaluations related to the self. For example, feeling happier for having enough to eat, feeling proud of success, feeling proud of a promotion, etc. (Izard, 1977; Lazarus, 1991). Thus, self-transcendent positive emotions increase the focus on others, decrease the focus on the self, and encourage greater connection with others than general positive emotions.

Self-transcendent positive emotions are fundamental to human sociality and have important social functions (Ekman, 2016). Self-transcendent positive emotions can help individuals form lasting commitments to kin, non-kin, and social collectives by fostering connections, commitments, and attachments to others (Stellar et al., 2017), and it also possesses the ability to help individuals initiate and maintain relationships with others (Shiota et al., 2007; Oveis et al., 2010; Gordon et al., 2012). And among the emotions most likely to trigger connection with the outside world are those associated with states of awe, such as awe, moral elevation, admiration, and inspiration (Van Cappellen et al., 2013; Stellar et al., 2017). And Moreton et al. (2019) has demonstrated the facilitating effect of moral uplift on connection with nature. Of the remaining positive emotions of self-transcendence, awe and gratitude are the ones that most stimulate an individual’s broad sense of connection with others (Moreton et al., 2019). In contrast to awe, gratitude, which also involves making external evaluations, differs significantly from the emotions associated with the state of awe, Haidt (2003) argue that awe evolved from social emotions that promote group cohesion, involves other-centered evaluations (Stellar et al., 2017), and does not require the experiencer of the emotion to go deeper into the situation to be felt. Gratitude, on the other hand, as an outwardly intense, self-centered emotional experience, requires a situation that is relevant to the person experiencing the emotion in order to trigger it. This study will therefore explore the effects of gratitude on promoting nature connection and pro-environmental tendencies.

### Gratitude and Pro-sociality

Gratitude has been a focus of research in the field of psychology in recent years (Kong et al., 2015; Williams and Bartlett, 2015; Bartlett and Desteno, 2016). Gratitude, also known as gratitude or feeling, is a positive emotion that individuals experience when they receive favors from others (McCullough et al., 2002). Trait gratitude refers to the fact that a person has a high sense of gratitude and will easily and frequently experience gratitude (Wood et al., 2008). It is a stable and implicit gratitude and a static expression that reflects individual differences State gratitude refers to the immediate emotional experience of individuals after they are helped by others, namely Gratitude Emotion (Emmons and Shelton, 2002), is a kind of immediate and explicit gratitude emotional experience, is a dynamic expression of gratitude, reflecting the difference of state. If the situation can just trigger gratitude, it will trigger individual gratitude emotion at the moment (Emmons and Kneezel, 2005; Froh et al., 2010), state gratitude represents one aspect of gratitude. Therefore, in this study the main focus was on the emotional state of gratitude.

McCullough et al. (2001) and others argue that gratitude arises from the perception that one person benefits from another’s costly, intentional, and voluntary actions and is a moral and pro-social emotion. Many studies have indicated that inducing gratitude promotes positive interpersonal relationships (Algoe et al., 2008; Duprey et al., 2020) and that it can enhance an individual’s sense of well-being, facilitate interpersonal interactions, and build and strengthen social relationships (Emmons et al., 2003; Yu and Xia, 2013) and supports that people who prioritize social relationships will benefit more from gratitude than those who do not (Boehm et al., 2011). During food sharing in chimpanzees, researchers have observed some basic forms of gratitude toward unrelated animals that have previously groomed them (Bonnie and de Waal, 2004). The experience and expression of gratitude strengthens reciprocity between non-kin, which over time can benefit individuals and encourage more cohesive groups (Trivers, 1971).

All the positive outcomes that gratitude promotes are due to the two main functions that gratitude has and the fact that gratitude is associated with pro-social personality traits. Gratitude can motivate individuals to reciprocate (Smyth, 2017), namely, gratitude has the function of motivating others to appreciate and promoting social connection, that is, gratitude may be one of the motivations behind reciprocal altruism (Trivers, 1971). The reinforcing function of gratitude is as a reinforcer of moral behavior (Emmons et al., 2001); expressing gratitude for someone’s pro-social behavior makes the giver feel valued and work harder to demonstrate pro-social behavior in the future, thus making gratitude a well-suited emotion to express (Gallup, 1998). This is usually motivated by (a) egoistic motives and (b) social conformity motives. Some people actively engage in pro-social behavior in part because they find expressing gratitude and other types of social identity to be highly reinforcing (Eisenberg et al., 1991). It has been suggested that grateful people have higher levels of agreeableness (Saucier and Goldberg, 1998), and that subjects who are induced to express gratitude have a stronger tendency to help others than other positive emotions (Grant and Gino, 2010) and behave more prosocially in subsequent interactions with others (Bartlett and DeSteno, 2006; Stellar et al., 2017), which somewhat increases the likelihood that individuals will exhibit pro-social behavior toward the giver in the future (McCullough et al., 2008), an effect that can last for months (Algoe et al., 2008) and is present in a variety of relationships (e.g., couples strangers; Gordon et al., 2012). Furthermore, keeping a weekly gratitude journal increases people’s sense of connection with others and reported pro-social tendencies compared to controlled activities (Emmons et al., 2003). These findings are consistent with earlier theoretical accounts of gratitude, suggesting that gratitude creates more cohesive and better functioning groups (Smith, 1790/1976). It has been found that people with high levels of gratitude are likely to appreciate and enjoy positive relationships in their lives and therefore may receive more peer support (Lyubomirsky et al., 2005), financial giving (Rind and Bordia, 1995) and help (Grant and Gino, 2010) from social resources. Latino American college students perceive the presence of experiences and expressions of gratitude as more welcoming than their peers (Corona et al., 2020).

There is an interconnection between connection to nature and connection to others, self-transcendent positive emotions promote social connection, gratitude is also strongly associated with being pro-social, and connection to nature is a strong predictor of environmental protection. It is therefore hypothesized that through self-transcending positive emotions and connection to nature, gratitude will increase the willingness to participate in environmental protection and the willingness to sacrifice for the environment.

Unlike gratitude emotions, recreational emotions do not influence one’s own behavior by witnessing the behavior of others, nor do they trigger a widespread sense of connectedness with others at a distance (Van Cappellen et al., 2013). Recreational emotions belongs to general positive emotion, which can only arouse the individual’s internal emotion, such as happiness and happiness. Therefore, in order to control for the effects of general positive affect, we followed many previous studies (Silvers and Haidt, 2008; Schnall et al., 2010) and added a recreational group to investigate the stronger effects of self-transcendent positive affect in promoting connectedness to nature and pro-environmental tendencies. This study used the Connection to Nature Scale to assess the emotional connection between self and nature (Mayer and Frantz, 2004; Tam et al., 2013) and three scales, the Nature into Self Scale, the Holistic Identity-Nature Subscale, and the Human Nature of Identity for All Inclusion Subscale, to assess whether the facilitative effects of gratitude on connection to nature extend to the cognitive overlap between self and nature.

The present work in this study aims to investigate the effects of gratitude on connectedness to nature, willingness to participate in environmental protection and willingness to sacrifice for the environment. We therefore divided the subjects into a gratitude group with self-transcending positive emotions, a recreation group with general positive emotions, and a control group with no emotion elicitation. Our hypothesis was that subjects who induced gratitude would report higher levels of connection to nature, willingness to participate in environmental protection, and willingness to sacrifice for the environment than the other two groups.

## Materials and Methods

### Participants and Procedure

For this study, we recruited a total of 988 undergraduates from Northwest Normal University in China to fill out an online questionnaire, and data from 98 subjects were excluded due to missing or identical answers on all options, resulting in 890 valid questionnaires (77% female, Mage = 20.16 years, SD = 2.04, NG = 250; NA = 360; NC = 280). Where this sample size calculation was based on a previous study by Moreton et al. (2019) with some additions to investigate the effect of gratitude on the outcomes of interest. All Participants volunteered to participate in the study and received a small gift as payment for completing the questionnaire.

### Instruments

#### Story Materials

The story materials were five gratitude stories, 20 amusement stories, and five science stories collected on the internet. The final one gratitude story, five amusement stories, and one neutral story were selected through the evaluation of 30 experts. The selection criterion for the gratitude story material was the perceived level of gratitude, and the 30 experts were asked to read each story and rate the perceived gratitude on a five-point scale, and finally select the story with the highest average rating among the five stories as the gratitude story material; the selection criterion for the amusement story material was the perceived level of amusement, and the experts were asked to rate the amusement level of each story on a five-point scale, and finally select the five stories with the highest average amusement The screening criterion for the neutral story material was the boredom of non-positive emotions, which was the same procedure as the first two materials, and the story with the highest mean value of boredom was selected. The gratitude story is about a grandmother who gives a bowl of noodles to a young boy under the kindly lie of the restaurant owner, after which the young boy wishes to return the favor to the grandmother on the same terms. The amusement stories are five short jokes, such as “The way to lose weight without dieting – eat garlic. The further away people are from you, the smaller they see you.” The control group story is a prospectus – the essential material and reference standard for a prospectus.

#### Intentions to Engage in Environmental Protection and Willingness to Sacrifice for the Environment

This section contained a total of 14 declarative sentences, 12 of which were about intentions to engage in environmental protection (α = 0.9) and 2 about willingness to sacrifice for the environment (α = 0.8). The material was selected from Moreton et al.’s (2019) study. For example: “Find ways in which some items can be reused,” “Buy reusable products,” “I am willing to pay a higher price for many goods and services in order to protect the environment,” “I am willing to accept a lower standard of living in order to protect the environment.” After reading each sentence you were asked to answer the question “How likely do you think you are to engage in this behavior in the future.” Responses were given on a 5-point Likert scale (1 = strongly disagree, 5 = strongly agree). The higher the score, the stronger the intentions to engage in environmental protection or sacrifice for the environment.

#### Connectedness to Nature Scale

Connectedness to Nature Scale was developed by Frantz et al. (2005) and revised by Li and Wu (2016) into a Chinese version, which consists of 14 items, include (1) I often feel integrated with nature. (2) Nature is my home. (3) I recognize and appreciate the wisdom of other creatures on earth. (4) I often feel separated from nature. (5) When I think about the meaning of life, I think I am a part of the life cycle of nature. (6) Animals and plants often give me a sense of intimacy. (7) The earth belongs to me, and I also belong to the earth. (8) I know very well how my behavior will affect nature. (9) I often feel that I am part of the web of life in nature. (10) Both human beings and other living beings have the same “way of endless life.” (11) Trees are a part of the forest, and I am also a part of nature. (12) I think I’m at the top of the natural pyramid. (13) In nature, I feel very small, as insignificant as flowers and trees. (14) My personal happiness has nothing to do with the quality of nature. This scale’s internal consistency reliability was 0.783; the test-retest reliability was 0.901; the Compatibility validity was 0.491.

A five-point Likert scale was used (1 = strongly disagree, 5 = strongly agree). Higher scores indicated a higher level of connection to nature (α = 0.84).

#### Emotion Scale

Eight self-transcendent positive emotions (*inspiration, love, feeling moved, respect, admiration, awe, gratitude, and humility*), five non-self-transcendent positive emotions (*joy, happiness, amusement, pride, and pleasure*), and five negative emotions (*anger, fear, shame, boredom, and sadness*) were included. The self-transcendent positive emotions were sampled from Moreton et al.’s (2019) study and the remaining emotions were sampled Van Cappellen et al. s’(2013) study. The 7-point Likert-type scale (1 = Not at all, 7 = Extremely) was used. Higher scores indicate stronger emotional feelings experienced. We selected the following three types of emotions to test the validity of the story material and to examine the role of self-transcendental positive emotions in promoting connectedness with nature (α = 0.88).

#### Self-Nature Overlap Scale

**FIGURE 1:**
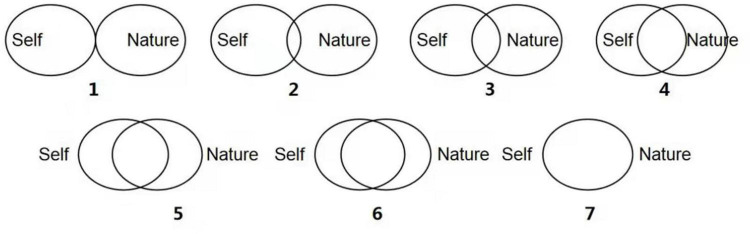
Inclusion of nature in self scale (INS).

The overlap between self and nature was measured by the Inclusion of Nature in Self Scale and the Allo-Inclusive Identity-Nature Subscale. The Inclusion of Nature in Self scale was selected from Schultz’s (2002) study and was represented by a picture containing seven images of two circles with different degrees of overlap, representing the degree to which the participant felt their perception of overlap with nature, and numbering their images (1 = least overlap, 7 = most overlap). The more overlap between the circles, the stronger the overlap between oneself and nature (see Figure 1). In the Inclusion of Nature in Self Scale, the question was designed to measure the participant’s feelings at the moment: “Please select the image below that best describes your connection to nature.” The higher the score, the greater the overlap between the participant’s perception and nature. The Allo-Inclusive Identity-Nature Subscale was selected from a study by Leary et al. (2008). This scale assesses the degree of overlap between oneself and wildlife. For example, “Please select the image below that best describes your connection to wildlife.” The participant selects the image that best represents their feelings at the moment (1 = least overlap, 7 = most overlap). The higher the score, the higher the degree of overlap between the participant’s perception and the animal. The material is similar to the Inclusion of Nature in Self Scale.

#### The Allo-Inclusive Identity Humanity-Subscale

The Allo-Inclusive Identity Humanity-Subscale represents the extent to which the self-overlaps with others. The scale assesses the extent to which one overlaps with a range of different people, from close people (e.g., “your best friend of the same sex”) to distant people (e.g., “strangers”). Higher scores indicate a higher degree of overlap with others (α = 0.76). The material is similar to that of the Inclusion of Nature in Self Scale.

### Procedure

Participants were first randomly assigned to each of three groups to watch a text story designed to induce gratitude, amusement, or boredom. At the end of the story, participants responded to eight positive emotions of self-transcendence, five general positive emotions and five negative emotions that they felt after reading the material. We used the eight self-transcendence positive emotion means as the self-transcendence positive emotion variable. This study expected self-transcendence mood levels to be higher in the gratitude condition. The Connectedness to Nature Scale was then completed and the mean values of the scale items were used to measure connection to nature. Participants then responded to their willingness to engage in 12 environmental behaviors and their willingness to sacrifice for 2 environmental behaviors, and finally completed the Inclusion of Nature in Self scale, the Allo-Inclusive Identity-Nature Subscale and the Allo-Inclusive Identity Humanity-Subscale in that order. The Self-Other Overlap measure was completed at the end of the survey after participants had completed all other measures.

### Statistical Analyses

**FIGURE 2:**
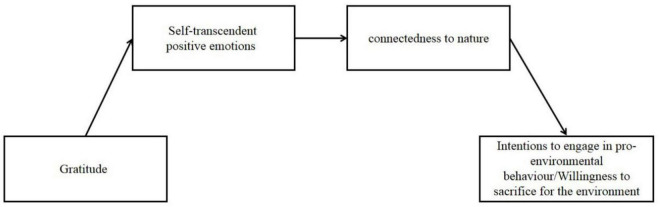
A conceptual model of gratitude indirectly influencing pro-environmental tendencies through positive emotions of self-transcendence and a connection to nature.

First, we conducted descriptive statistics based on Pearson correlation coefficients between nature-related variables and emotion variables (Table 1), then inferential statistics using a one-way ANOVA to examine differences between the three groups, and finally three sets of exploratory mediation analyses: one analyzed whether positive emotions of self-transcendence mediated the link between the gratitude emotion condition pair and nature, and the other two analyses extended this causal chain to include willingness to engage in environmental protection and willingness to make sacrifices for the environment (see conceptual model in Figure 2). The total, direct and indirect effects of these analyses are shown in Table 3, and the path coefficients are plotted in Figure 2.

**TABLE 1:** Correlations between nature-related variables and felt emotions.

	1	2	3	4	5	6
1. Connectedness to Nature	(0.84)					
2. Intentions to engage in environmental protection	0.58**	(0.90)				
3. Willingness to sacrifice for the environment	0.34**	0.69**	(0.8)	3		
4. The Inclusion of Nature in Self Scale	0.41**	0.29**	0.19**	(NA)		
5. The Allo-Inclusive Identity-Nature Subscale	0.28**	0.28**	0.20**	0.51**	(NA)	
6. The Allo-Inclusive Identity Humanity Subscale	0.04	0.17**	0.23**	0.29**	0.37**	(0.76)
4. Inspiration	0.14**	0.25**	0.26**	0.07*	0.09**	0.22**
5. Love	0.10**	0.22**	0.24**	0.08*	0.08*	0.19**
6. Feeling Moved	0.08*	0.21**	0.22**	0.07*	0.07*	0.18**
7. Respect	0.14**	0.22**	0.20**	0.09**	0.08*	0.14**
8. Admiration	0.15**	0.23**	0.22**	0.07*	0.08*	0.18**
9. Awe	0.13**	0.24**	0.24**	0.07*	0.10**	0.19**
10. Gratitude	0.12**	0.23**	0.24**	0.07*	0.09*	0.18**
11. Humility	0.13**	0.24**	0.28**	0.08*	0.10**	0.20**
12. Total self-transcendent positive emotions	0.13**	0.25**	0.26**	0.08*	0.09**	0.20**
13. Happiness	0.11**	0.23**	0.24**	0.06	0.06	0.18**
14. Amusement	0.18**	0.24**	0.21**	0.12**	0.09**	0.16**
15. Pride	0.09**	0.25**	0.30**	0.06	0.12**	0.21**
16. Joy	0.19**	0.24**	0.21**	0.11**	0.08**	0.14**
17. Pleasure	0.19**	0.24**	0.22**	0.10**	0.06**	0.14**
18. Anger	−0.19**	0.03	0.14**	−0.07*	–0.02	0.19**
19. Fear	−0.18**	0.03	0.16**	–0.06	–0.02	0.17**
20. Shame	−0.15**	0.04	0.15**	–0.06	–0.002	0.16**
21. Boredom	–0.04	–0.004	0.05	–0.06	–0.004	0.06
22. Sadness	−0.18**	0.02	0.14**	−0.07*	0.002	0.17**

*p < 0.05, **p < 0.01, Cronbach’s alphas in parentheses.

## Results

### Descriptive Statistics

We analyzed the correlation between nature-related variables and emotions using Pearson’s correlation coefficient (Table 1). The results show that two variables, contact with nature and willingness to sacrifice for the environment, are correlated with all emotion variables (except boredom), while willingness to participate in environmental protection only shows a correlation with positive emotions. All self-transcendent positive emotions and general positive emotions were moderately correlated with connection to nature, willingness to participate in environmental protection and willingness to sacrifice for the environment, and less correlated with self-other and self-nature cognitive overlap.

### Inferential Statistics

Analysis of emotion-related variables revealed that subjects in the gratitude group reported higher levels of inspiration, love, touching, respect, admiration, awe, gratitude and humility than subjects in the other conditions, a result that supports the validity of inducing gratitude. The Scheffe *post hoc* test revealed that (Table 2), compared to the recreation group (*p* < 0.001, *d* = 1.91) and the control group (*p* < 0.001, *d* = 1.27), the gratitude group had higher scores for self-transcendent positive emotions, and there was also a significant difference between the recreation and control groups (*p* = 0.003, *d* = 0.68). To test the validity of the recreational material, the control group had lower recreational mood scores compared to the gratitude group (*p* < 0.001, *d* = 0.60) and the recreational group (*p* < 0.001, *d* = 0.47), and there was no significant difference between the gratitude and recreational groups on the recreational mood scores (*p* = 0.15, *d* = 0.12), suggesting partial validity of the recreational material. A possible explanation for this is because both gratitude and recreation are positive emotions, and gratitude is actually a happy emotion expressed toward the giver.

**TABLE 2:** Effects of condition on mean score of outcomes.

	Gratitude group	Amusement group	B	F	P	η^2^
1. Connectedness to Nature	4.19 (0.52)	4.17 (0.64)	4.07 (0.62)	3.22	0.04*	0.007
2. Intentions to engage in environmental protection	3.63 (0.74)	3.46 (0.75)	3.46 (0.66)	4.87	0.008**	0.01
3. Willingness to sacrifice for the environment	3.33 (0.96)	3.10 (1.00)	3.20 (0.82)	4.77	0.009**	0.01
4. The Inclusion of Nature in Self Scale	5.13 (1.51)	5.13 (1.60)	4.95 (1.67)	1.14	0.32	0.007
5. The Allo-Inclusive Identity-Nature Subscale	4.07 (1.61)	3.97 (1.66)	3.91 (1.71)	0.61	0.54	0.001
6. The Allo-Inclusive Identity Humanity Subscale	3.46 (1.23)	3.33 (1.21)	3.39 (1.20)	0.88	0.42	0.002
7. Inspiration	5.11 (1.54)	2.59 (1.82)	3.50 (1.69)	162.66	< 0.001**	0.27
8. Love	5.46 (1.42)	2.46 (1.76)	3.23 (1.70)	250.17	< 0.001**	0.36
9. Feeling Moved	5.54 (1.45)	2.35 (1.74)	3.18 (1.68)	286.70	< 0.001**	0.39
10. Respect	5.69 (1.38)	2.70 (1.89)	4.11 (1.81)	219.57	< 0.001**	0.33
11. Admiration	5.42 (1.47)	2.63 (1.88)	3.86 (1.77)	191.53	< 0.001**	0.30
12. Awe	5.11 (1.64)	2.39 (1.76)	3.65 (1.76)	184.27	< 0.001**	0.29
13. Gratitude	5.49 (1.42)	2.44 (1.81)	3.34 (1.67)	252.49	< 0.001**	0.36
14. Humility	4.97 (1.64)	2.47 (1.79)	3.68 (1.70)	156.53	< 0.001**	0.26
15. Total self-transcendent positive emotions	5.35 (1.31)	2.50 (1.65)	3.57 (1.49)	262.14	< 0.001**	0.37
16. Happiness	5.54 (1.43)	2.61 (1.81)	3.30 (1.72)	233.14	< 0.001**	0.34
17. Amusement	4.26 (1.99)	4.02 (2.02)	3.15 (1.67)	25.60	< 0.001**	0.05
18. Pride	4.74 (1.86)	2.25 (1.68)	3.28 (1.72)	149.05	< 0.001**	0.25
19. Joy	4.82 (1.75)	3.79 (2.01)	3.20 (1.66)	52.68	< 0.001**	0.11
20. Pleasure	5.02 (1.77)	3.81 (2.07)	3.20 (1.72)	63.85	< 0.001**	0.13
21. Anger	2.32 (1.94)	2.03 (1.56)	2.47 (1.55)	5.87	0.003**	0.01
22. Fear	2.33 (1.90)	1.90 (1.52)	2.45 (1.57)	10.11	< 0.001**	0.22
23. Shame	2.28 (1.93)	2.02 (1.55)	2.35 (1.52)	3.57	0.03*	0.008
24. Boredom	2.40 (1.91)	3.48 (2.14)	3.25 (1.79)	23.08	< 0.001**	0.05
25. Sadness	2.54 (1.97)	1.89 (1.54)	2.38 (1.69)	12.67	< 0.001**	0.03

*p < 0.05, **p < 0.01. Standard deviations in parentheses.

**TABLE 3:** Mediating effects values and confidence intervals under the gratitude condition.

	*Effectsize*	Boot SE	95% confidence interval		The proportion of effector

		Lowerlimit		High limit	
A mediation with a Connectedness to nature as the dependent variable					
Total effect	0.44	0.07	0.30	0.57	100%
Direct effect	0.23	0.10	0.02	0.43	52.27%
Indirect effect	0.21	0.07	0.08	0.35	47.73%
A chain intermediary with intentions to engage in pro-environmental behavior as the dependent variable					
Total effect	0.28	0.09	0.11	0.46	
Direct effect	–0.10	0.10	–0.29	0.10	
Total indirect effect	0.38	0.07	0.25	0.52	100%
Ind1: Gratitude→Self-transcendent positive emotions→Intentions to engage in pro-environmental behavior	0.19	0.07	0.05	0.32	50%
Ind2: Gratitude→Self-transcendent positive emotions→Connectedness to nature→Intentions to engage in pro-environmental behavior	0.09	0.04	0.03	0.18	23.68%
Ind3: Gratitude→Connectedness to nature→Intentions to engage in pro-environmental behavior	0.10	0.04	0.03	0.19	26.32%
A chain intermediary with willingness to sacrifice for the environment as the dependent variable					
Total effect	0.20	0.08	0.04	0.35	
Direct effect	–0.01	0.10	–0.20	0.18	
Total indirect effect	0.21	0.07	0.08	0.34	100%
Ind1: Gratitude→Self-transcendent positive emotions→Willingness to sacrifice for the environment	0.12	0.06	–0.02	0.23	57.14%
Ind2: Gratitude→Self-transcendent positive emotions→Connectedness to nature→Willingness to sacrifice for the environment	0.04	0.02	0.01	0.10	19.05%
Ind3: Gratitude→Connectedness to nature→Willingness to sacrifice for the environment	0.05	0.03	0.01	0.11	23.81%

*All values are rounded to the nearest two decimal places.*

Analysis of the nature-related variable Scheffe’s *post hoc* test revealed that the recreation (*p* = 0.04, *d* = 0.16) and gratitude (*p* = 0.02, *d* = 0.21) groups scored higher on the level of connection to nature compared to the control group, with no significant difference between the recreation and control groups (*p* = 0.76, *d* = 0.03). The Gratitude group scored significantly higher than the Recreation group (*p* = 0.01, *d* = 0.23) and the Control group (*p* = 0.01, *d* = 0.24) on willingness to participate in environmental protection, but there was no significant difference between the Recreation and Control group scores (*p* = 0.91, *d* < 0.001). The gratitude group scored significantly higher than the recreation group (*p* = 0.003, *d* = 0.24) and the control group (*p* = 0.07, *d* = 0.16) in terms of willingness to sacrifice for the environment, but there was no significant difference between recreation and control group scores (*p* = 0.19, *d* = 0.11). Finally, the grouping condition did not have an effect on self-natural cognitive overlap and self-other cognitive overlap, in line with Moreton et al.’s (2019) study.

### Mediation Analyses

In the first mediation analysis, we used gratitude as the independent variable, scores on the connection to nature scale as the dependent variable output, and the total self-transcendent positive emotion variable as the mediating variable input, using PROCESS in SPSS (Hayes, 2012) for the mediation analysis, using model 4 with 5000 bootstrap samples and 95% confidence intervals. As can be seen in Table 3 the gratitude condition has a significant indirect effect on connection to nature through positive emotions of self-transcendence and plays a partially mediating role. Then in the latter two sets of mediation analyses we used gratitude as the independent variable, willingness to participate in environmental protection or willingness to sacrifice for the environment as the dependent variable, the total self-transcendent positive emotion variable as the first mediating variable and the score on the connection with nature scale as the second mediating variable using model 6 (5000 bootstrap samples), which allowed for multiple mediators to be tested consecutively. As can be seen in Table 3, there is a significant indirect effect of gratitude on willingness to participate in environmental protection/willingness to sacrifice for the environment through self-transcendent positive emotions and connection to nature, and a fully mediating effect.

## Discussion

Previous research has found that connection to nature is associated with positive emotions (Capaldi et al., 2014). However, little attention has been paid to whether specific positive emotions can increase connection to nature, with only Moreton et al. (2019) focusing on the contribution of morally uplifting emotions to connection to nature. The present study therefore aimed to test the hypothesis that gratitude increases connection to nature, and the results largely supported this hypothesis. Compared to the control group, subjects who received gratitude induced emotions reported significantly higher connection with nature scores; while no significant differences were found in connection with nature scores compared to the recreational group. Possible explanations for this are that both gratitude and recreation are positive emotions, and positive emotions are inherently conducive to connecting with nature.

These findings have both theoretical and practical implications. There is growing evidence that the future of humanity is inextricably linked to nature, and Bragg (1996) proposes the theory of the ecological self, which argues that human well-being and the well-being of nature are intertwined, so that happiness is experienced through a spiritual interconnection with all things. On the one hand, connection to the natural world is beneficial to people’s survival. Mental health is strongly linked to a sense of natural connection (Howell et al., 2011; Cervinka et al., 2012), and those who are emotionally resonant with the natural world tend to function well psychologically (Trigwell et al., 2014), reducing the incidence of mental illness (e.g., depression, None., 2016). Natural connection has also demonstrated positive relationships with dimensions of well-being in terms of personal growth, autonomy and life purpose (Nisbet et al., 2011; Zelenski and Nisbet, 2014). On the other hand, an increased connection to nature can also have beneficial effects on the development of the natural world; Schultz (2001) found that participants who incorporated nature into their concept had more environmentally friendly beliefs and engaged in more pro-environmental behaviors. The current study found gratitude to have a fully mediating effect on willingness to engage in environmental protection/sacrifice for the environment through self-transcending positive emotions and connection to nature, whereas Moreton et al.’s (2019) study found only partial mediation. This result suggests a strong, albeit exploratory, effect of gratitude on the promotion of connection to nature and enhanced pro-environmental tendencies, but these effects also suggest that self-transcendent positive emotions may lead to environmental conservationism through increased connection to nature. These effects are consistent with previous research linking nature and pro-environmental behavior (Mackay and Schmitt, 2019).

However, while the current study found that subjects in the gratitude condition reported higher scores on connection with nature and greater willingness to engage in environmental protection and sacrifice for the environment, there were no subgroup differences in scores on the overall sameness-nature subscale, or the nature-inclusion self scale. This suggests that emotions only promote emotional connection to nature, but not cognitive connection to nature, i.e., emotions related to self-transcendence and connection to nature do not play a role in bringing cognitive connections closer. However the cognitive aspect may be related to self-transcendence as a Meaning-Mindset of a life attitude (Wong et al., 2021). Furthermore, although there was evidence that connection to nature promotes connection to others, there were no subgroup differences in scores on the Human Nature of Identity subscale for inclusive people. A possible conjecture for this result is that gratitude, as an emotion, is more likely to affect emotional rather than cognitive connections. However although no significant group differences in scores emerged, scores in the gratitude condition were still higher, so perhaps this will change when the sample size is expanded.

## Limitations and Future Directions

The main limitations of this study are as follows: firstly, as the choice of emotion elicitation method was not the most effective, the results were not consistent with previous studies. It has been shown that music and video are the most effective of the various methods of emotion elicitation and are relatively homogeneous (Green et al., 2010). Therefore, future research should be conducted using the more evocative methods of music and video. At the same time the evocative effect on the entertainment story material was not significant, therefore future research should focus on the distinction of commonalities between positive emotions and self-transcending positive emotions. Emotional control of the subjects before the start of the formal experiment was essential to effectively control for differences in the subjects’ emotions and explain the differences between the different groups. Secondly, the data collection process for this study was conducted by filling out an electronic questionnaire, therefore the effectiveness of the questionnaire could not be guaranteed and there were more uncontrollable additional variables. Future studies could be conducted using one-to-one fixed sites, or question and answer to better control for additional variables. Thirdly, the subjects in this study were selected from a sample of university students, who are perhaps not as good as adolescents or children in terms of perception of the environment and empathy for it. Future studies could be attempted using primary and secondary school subjects. Fourth, among the self-transcendence-related positive emotions are awe and empathy, and they could be studied to replicate the relationship between self-transcendence-related positive emotions on connection to nature. Perhaps non-self-transcendence positive emotions would have the same effect and could be explored in future studies. Finally, Lamb (1996) argues that the importance humans place on nature will have an impact on how humans perceive their connection to nature, meaning that there is variability in the degree to which nature is perceived. In other words, if a person desires to be connected to nature, he or she may have a more connected view of nature and humans than someone who desires isolation (Vining et al., 2008). Thus variability in an individual’s psychological arousal can also affect the degree of connection to nature. Future research could explore this aspect.

## Conclusion

Humans are closely related to nature, and human connection and human connection to nature are closely related. Supporting this, current research suggests that the induction of self-transcending positive emotions that are closely related to others can facilitate an individual’s emotional connection to the natural world. Individuals in a state of gratitude have a higher level of connection to nature and are able to elicit stronger environmental emotions in individuals, but remain uncertain about integrating the self into nature, i.e., the self as a part of nature. Future research could continue to explore the potential conditions between self-transcending positive emotions and nature connection and environmental protection.

## Data Availability Statement

The original contributions presented in the study are included in the article/supplementary material, further inquiries can be directed to the corresponding author.

## Ethics Statement

The studies involving human participants were reviewed and approved by the Scientific and Research Ethics Committee of the School of Psychology, NWNU. The participants provided their written informed consent to participate in this study.

## Author Contributions

LC contributed to the conceptualization, funding acquisition, project administration, supervision, and writing—review and editing. JL contributed to the investigation, methodology, and English writing—original draft. LF contributed to the data curation, visualization, and writing—original draft. CG and YC contributed to the formal analysis and validation. All authors have read and agreed to the published version of the manuscript.

## Conflict of Interest

The authors declare that the research was conducted in the absence of any commercial or financial relationships that could be construed as a potential conflict of interest.

## Publisher’s Note

All claims expressed in this article are solely those of the authors and do not necessarily represent those of their affiliated organizations, or those of the publisher, the editors and the reviewers. Any product that may be evaluated in this article, or claim that may be made by its manufacturer, is not guaranteed or endorsed by the publisher.
